# Temperament Differences

**Published:** 1882-04

**Authors:** 


### TEMPERAMENT DIFFERENCES.

Angel or angel fallen, fills the description of a woman for her life time. For a month before marriage, and a month after death, men (whether many or most, deponent saith not,) regard their wives as angels; all between, angels fallen; the only difference being, that men are angels never. Young ladies before marriage, and widows, strive to make themselves pleasing, and they succeed, the latter especially. After awhile the decree of divorce reads, "on account of incompatibility of temper." But they pleased one another once, and could do it again, and to the end; only the effort is wanting, that tells the whole story. In these times, when a degraded press, under the prostituted plea of "liberty," finds a mine of wealth, and consequently gloats over a "divorce case," whether of mechanic or millionaire, whether of the "boards" or the "pulpit," laying before our wives and daughters, morning after morning, the bought-up testimony of outsiders, and the filthy rehearsals of impossible facts by malicious or time serving menials, when these things are so, it becomes those of us who are parents, to consider seriously if something cannot be done to devise safeguards, at least in individual instances, against the calamity of the ought-to-be sacred precincts of the family, invaded by impertinent reporters and lawyers, not less worthy of public execration, who, forgetting that they themselves are husbands and fathers, too often, in the halls of justice, indulge in obscene ribaldries, and disgusting probings; not for truth's sake, but for purposes of sarcasm and of personal abuse, as if right was not to be obtained, without these degrading and infamous aids.

There is a great deal of rank atheism in some of the teachings of phrenology, possibly confined to its blind teachers, in that crime is excused virtually, on account of the "temperament," or "physical conformation." We admit that as far as phrenology teaches and encourages us to rectify these deformities, it is well, but by throwing out intimations, that to a certain extent, crime is excusable on these accounts, making it a moral insanity, it uproots all law, human and Divine, and moral anarchy reigns universal.

We firmly believe, and most unhesitatingly avow, that a sound christianity, early instilled, will uproot this whole matter, and make domestic life akin to angelic communion. The fundamental teaching, the great practical idea of Bible religion, is expressed in two words by its Divine Author, "Deny Thyself." Taking a wider range, it means self control. In its application to all that is desirable, "I can't" is not in the vocabulary of a man, of a christian. That an efficient early self control is possible, is proven in the fact of its practice before marriage; there is nothing in that ceremony which makes it less practicable. The power exists, only the effort is wanting. Let all parents then, let all who are about to be married, let all the married, remember that the inculcation, the practice of self control, is the key stone of practical religion, it is the panacea for domestic disquietude, the entrance door of a heaven on earth, and a brighter heaven in the skies.

On the other hand, let those remember, whether husbands or wives, that the indisposition to make this effort, is not only a want of all religion, it is a want of all nobility, it is beast-like; it demonstrates the want of gentle blood, and the presence of a nature essentially low, one which no improved condition of outward circumstances would ever elevate, but which on the contrary, would be fed by them, and the aids to elevation would be only prostituted to purposes of a deeper degradation. In plainer phrase, the man or woman who will excuse an acknowledged short-coming, by saying "I would do better if I were better off," give me this, that or the other which I have not now, and I will show you that I will be a different being, that person is yet "in the gall of bitterness, and in the bond of iniquity," and needs the pity and the prayer of all good men.

### WEAKLY YOUTHS.

WITHIN one week, three persons have complained that their lives have been made lives of suffering, by the ignorance of parents, thus: They grew up rapidly, almost as tall at sixteen as at mature age. The rapidity of their growth was attended with great debility, while the parents judging of the ability to work by the size, required more of them than they were able to perform, and a strain was imposed upon their constitutions, which made them a wreck after; not indeed destroying life, but leaving the body a shell, and all its functions so impaired, as to their capabilities, that none of their work was well performed, resulting in disease of the whole system, making life a torture, and in one case we know of, there is a never failing reprehension of parental memory.

Persons who are healthy and hearty themselves, do not know how to sympathize with a rapidly growing child, and their complaints of weariness are unheeded, blamed or scolded at. To all parents then, especially to farmers and mechanics, we give the advice, when a child has grown up rapidly, impose but little labor, and that, never violent nor long protracted; it should be light, short, steady, not by fits and starts, never drive, always encourage, and when they go to bed at a regular, early hour, let them have all the sleep they will take, never allow them to be waked up, let nature do that, and she will do it regularly, and in due time.

---

### WALK SOFTLY.

THE tiniest pebble thrown sea-ward from the beach, causes a wavelet, whose influences are felt for unnumbered leagues out upon old ocean's bosom. The softest whisper excites vibrations in the atmosphere around us, which cease not this side the boundless ether; so the act or thought of an immortal man, however insignificant, may color a lifetime, may leave influences which shall not cease, until time shall be no longer; influences for good or ill, to millions of immortals like himself, for unending ages. These things being so, it would seem that every act should be a felt responsibility, and every thought a prayer. Let us all walk softly then, or at least with a motive and a wish for good.

A crust of bread thrown thoughtlessly by a fellow student, made Prescott, in a measure, sightless for near half a century. An ill-timed jest has severed many a warm friendship, and planted bitterness for a lifetime, where ought to have welled up the warmest, and purest, and loveliest springs of our nature. Many a time and oft, has a frown, a harsh word, an unfeeling or contemptous gesture, crushed resolves forever, which were budding to a new and changed and better life. Reader, let us all walk softly then by day and by night, at home and abroad, inasmuch as for every step in life, we must give account at the judgment.

